# Corporate Welfare and Parenting Self-Regulation as Protective Resources Against Stress and Quiet Quitting: A Moderated Mediation Model Across Mothers and Fathers

**DOI:** 10.3390/bs16050743

**Published:** 2026-05-11

**Authors:** Sebastiano Rapisarda, Damiano Girardi, Jessica Pileri, Alessandra Falco, Laura Dal Corso

**Affiliations:** 1 Department of Philosophy, Sociology, Education, and Applied Psychology, University of Padua, 35131 Padua, Italy; sebastiano.rapisarda@unipd.it (S.R.); damiano.girardi@unipd.it (D.G.); alessandra.falco@unipd.it (A.F.); 2 Department of Pedagogy, Psychology, Philosophy, University of Cagliari, 09127 Cagliari, Italy; jessica.pileri93@unica.it

**Keywords:** Perceived Corporate Welfare Scale, parental self-efficacy, stress, quiet quitting, parenthood, motherhood, fatherhood, well-being, HR policies, Job Demands-Resources

## Abstract

While parenthood is gratifying, it is also a significant life transition, filled with challenges and stressors that require ongoing psychological and behavioral adjustments. This study aims to: (1) verify the psychometric characteristics of the Perceived Corporate Welfare Scale (PCWS), and (2), in line with the Job Demands-Resources (JD-R) theory, investigate how corporate welfare and parenting self-regulation act as resources against perceived stress and quiet quitting. We further explored the mediating role of stress and the moderating effect of parenthood. A group of 788 Italian workers (43.5% non-parents; 29.6% moms; 26.9% dads) participated. The psychometric properties of the PCWS were tested using CFA. A moderated mediation model was estimated using Bootstrap methods (95% CI). The PCWS showed a robust one-factor structure with significant item loadings (>0.60) and satisfactory reliability (CR and AVE). Findings suggest that perceived corporate welfare and parenting self-regulation function as resources and are negatively associated with perceived stress. Perceived stress fully mediates the relationship between perceived corporate welfare and quiet quitting and partially mediates the relationship between parenting self-regulation and quiet quitting. Crucially, parenthood moderates this relationship: fathers’ perceived stress has a stronger association with quiet quitting than mothers’ does. Consequently, the indirect effects are also stronger for fathers. This study provides a validated tool for monitoring perceptions of corporate welfare. The results suggest that personalized interventions and an organizational culture that values parenthood are key to sustaining well-being and long-term employee engagement.

## 1. Introduction

The current state of the labor market, heavily impacted by post-pandemic changes and the Industry 5.0 model, has prompted a reevaluation of organizational well-being as a key factor in a business’s attractiveness and sustainability (Ikenga & Van Der Sijde, 2024; Trstenjak et al., 2025; World Economic Forum, 2025). 

In Italy, the importance of work lies in its ability to improve workers’ quality of life. In fact, 88.2% of Italian workers believe that time dedicated to subjective well-being should be a universally recognized right, that organizational well-being is essential to work (87.4%), and that companies should be committed to promoting workers’ overall well-being (83.6%). Clearly, well-being has taken priority over work. In this context, corporate welfare is a significant step towards holistic well-being (CENSIS, 2026). 

Corporate welfare is a complex system of private, employer-based, or collective nature that provides goods, services, and benefits with a social purpose to employees in addition to monetary remuneration. It can be included in the transition from a traditional welfare state to a welfare society. In the former model, the state is the primary service provider, and the goal is to ensure a minimum standard of living for citizens unable to work and lacking the resources for sustenance. The latter is characterized by a multi-actor, multi-level governance structure, in which industrial relations actors assume a proactive role in social protection, aiming to provide a more flexible and customized response to people’s needs (De Falco, 2023; Olivelli, 2020). Since the 1990s, characterized by the financial sustainability crisis in the public sector and the emergence of new social risks (e.g., demographic aging, work–life balance, dependency), corporate welfare has emerged as an integrative and subsidiary pillar (Busacca & Pasian, 2025). To understand the legislative framework for corporate welfare in Italy, it is helpful to examine the tax incentive system. Articles 51 and 100 of the TUIR (Italian Tax Consolidated Text)^1^ are the cornerstone of this system. They define the conditions for tax exemption and for relief from social security contributions for goods and services intended for social purposes. Significant progress was made with the 2016 Stability Law (Law 208/2015)^2^ and the 2017 Budget Law (Law 232/2016)^3^, which expanded tax-exempt services and benefits for employees and their families, as well as the option to convert performance bonuses into welfare services.

Italian workers increasingly expect corporate welfare measures to be tailored to each individual. The importance of corporate welfare for attraction, retention, and productivity is evident: 71.6% of workers would choose a company with a well-designed corporate welfare plan if they had to switch jobs. Furthermore, 84.1% believe that corporate welfare improves motivation and productivity. In addition to the personalization, workers also value clear communication (88.9%) and ease of use (95.1%) of welfare measures. The most popular corporate welfare services are income support (86.2%), healthcare (76.8%), and psychological support (41.4%). The most desired services, however, are those related to family assistance, such as company nurseries (80.7%), assistance for the elderly and dependent persons (77.1%), and services for children and minors (68.6%) (CENSIS, 2026).

Therefore, corporate welfare can support all workers, especially parents. Parenthood is one of the most complex stages of life. It is often characterized by new challenges that may disrupt the balance between work and family life, with possible consequences for parents’ health (Saxbe et al., 2018). Working mothers face even more complex challenges. Rigid maternal beliefs often fuel these challenges. Social expectations impose standards of perfection difficult to reconcile with work. These beliefs can undermine the physical and mental well-being of working mothers, increasing the risk of negative outcomes such as emotional disinvestment or leaving the workforce (Rapisarda et al., 2024a; Thomason et al., 2015). Statistical data confirm the critical nature of this situation. An analysis of the employment situation in Italy reveals persistent gender inequality, which is exacerbated further by the presence of children. In single-income households, the distribution of labor market participation still appears to be anchored to traditional models: in 85% of cases, the man is the sole employed partner. The gender gap, which is around 15 percentage points in childless households, increases with the presence of children: the female employment rate falls to 59.9%, while the male rate rises to 87.3%. The gender gap reaches its critical peak when the age of the children is considered: the presence of at least one child under 5 brings the difference in employment rates between men and women to 34 percentage points, with mothers’ employment rate falling to 57.8% (Sviluppo Lavoro Italia, 2026). On the other hand, fatherhood is undergoing a redefinition. There has been a shift from the traditional breadwinner model to a more active and conscious involvement in caregiving practices. However, fathers often encounter cultural and organizational obstacles, including social stigma and fear of professional retaliation (i.e., fatherhood penalty). These obstacles can limit access to work–life balance measures and generate high levels of stress and low parental self-efficacy (Coltrane et al., 2013; Kvande & Brandth, 2020). 

Therefore, targeted policies such as flexible work schedules and family support services are not just tools for retaining staff; they are also levers for promoting inclusion and gender equality (Chung & Van Der Lippe, 2020). Therefore, it is essential that organizations adopt assessment tools that can identify workers’ real needs and perceptions. This transforms welfare from an administrative product into a relational process aimed at enhancing human capital. In this scenario, the following section presents the Job Demands-Resources theory (Bakker et al., 2023; Bakker & Demerouti, 2017), which serves as the theoretical foundation of our study. 

### 1.1. Job Demands-Resources Theory

The Job Demands–Resources (JD-R) theory (Bakker et al., 2023; Bakker & Demerouti, 2017) is a widely accepted theory of job design that describes how different characteristics of the work environment affect job performance by shaping employee well-being, including levels of burnout and work engagement (Bakker et al., 2023). Originally, the theory postulated two broad categories of working conditions: job demands and job resources (Demerouti et al., 2001). Since people tend to bring work-related stress home and family stress to work, especially nowadays when many workers are choosing to work remotely, the boundaries between these spaces are becoming even more blurred (Bakker et al., 2011; De Carlo et al., 2019; ten Brummelhuis & Bakker, 2012). Taking these complexities into account, more recent theorizations (Demerouti & Bakker, 2023) identify organizational, job, personal, and home demands. These are defined as aspects that require prolonged physical, cognitive, and/or emotional effort and are therefore associated with certain physiological and/or psychological costs. Organizational, job, personal, and home resources are defined as those aspects that have motivational potential, that are functional to the achievement of professional objectives, that regulate the impact of demands, and that stimulate learning and personal growth. Work demands (i.e., organizational and job) and nonwork demands (i.e., personal and home) interact such that the effects of each on health outcomes are exacerbated when the other demands are high. Similarly, work and nonwork resources interact such that the effect of each on motivational outcomes is exacerbated when the other is high. The authors also identify some regulatory strategies and different levels (organizational, leader, family, and individual) to buffer the unfavorable impact of demands from either domain on health-related outcomes and boost the positive impact of resources from either domain on motivational outcomes. Although integrating these aspects may increase the complexity of JD-R theory, it allows us to better understand that individual and organizational well-being does not depend solely on purely organizational factors but also on interactions with various domains of life.

### 1.2. Corporate Welfare 

Corporate welfare measures (e.g., flexible working hours, parenting support, accessible healthcare) and their perception are not just economic benefits. Perceived corporate welfare focuses on the effectiveness and quality of the plan itself rather than satisfaction with or awareness of the benefits. It evaluates whether measures are adequately communicated and implemented based on an analysis of employees’ specific needs, such as caregiving burdens, and continuously monitors their effectiveness. These perceptions are based on one’s own organization. Beyond monetary considerations, this perception encompasses an organization’s commitment to sustainability, closing the pay gap, promoting gender equity, and fostering a family-oriented culture. Rather than evaluating the fairness of the exchange, perceived corporate welfare focuses on non-monetary characteristics to ensure that resources are oriented toward the holistic care of the individual. By providing accessible, personalized, sustainable measures that address actual needs, the organization can transform welfare into a strategic resource that promotes individual and organizational well-being. It may be useful to distinguish the perception of corporate welfare from other dimensions, such as perceived organizational support (POS), organizational justice, employer attractiveness, and total reward satisfaction. As is well known, POS represents an employee’s overall belief that the organization values their contributions and cares about their well-being. This belief is fundamentally based on the norms of reciprocity and social exchange (Allen et al., 2003; Battistelli & Mariani, 2011; Eisenberger et al., 1986; Rhoades & Eisenberger, 2002). Organizational justice evaluates the fairness of treatment and resource allocation based on impartiality and reciprocity (Colquitt, 2001; Spagnoli et al., 2017). Employer attractiveness focuses on evaluating an overall compensation package, including an above-average salary, and is typically assessed relative to a hypothetical organization (Berthon et al., 2005; Caputo et al., 2023). Finally, total reward satisfaction reflects an employee’s overall satisfaction with the mix of monetary and non-monetary rewards received based on skills, performance, and market value (Hareendrakumar et al., 2021).

Therefore, perceived corporate welfare can be considered an organizational resource that promotes motivation, prevents burnout, and mitigates the negative effects of increased demands (Bakker et al., 2023; Dal Corso, 2024; Demerouti & Bakker, 2023). Giorgi et al. (2020) found that organizational orientation to employee welfare was negatively associated with higher exposure to negative workplace behaviors, such as workplace bullying. However, economic stress makes employees more sensitive to the treatment they receive from their organization during uncertain times, thereby reducing their perception of the organization’s orientation toward employee welfare. Dal Corso and Rapisarda (in press) highlighted that a positive perception of corporate welfare can improve working mothers’ occupational future time perspective and indirectly reduce quiet quitting. The authors also noted that perceived corporate welfare, as an organizational resource, promotes parental self-efficacy among working fathers and indirectly reduces work-family and family-work conflict. However, the effectiveness of a corporate welfare system depends not only on the variety of benefits provided but also on the transition from a welfare “product” logic to a welfare “process” logic (D’Angelo et al., 2020). Therefore, workers’ perceptions of personalization, communication, and the plan’s responsiveness to individual needs, including care burdens, are important (CENSIS, 2026). These perceptions nourish the psychological contract, promoting positive outcomes such as a more favorable occupational future time perspective (Dal Corso, 2024). Thus, it is essential to implement assessment measures that capture workers’ perceptions. Analyzing subjective needs and the meaning attributed to implemented measures allows welfare to become a real organizational resource. Adopting a person-centered measurement approach allows organizations to tailor welfare policies, ensuring that interventions are not merely formal but effectively promote sustainable health outcomes.

### 1.3. Parenting Self-Regulation 

While parenthood is a gratifying experience, it also represents a significant life transition filled with challenges and stressors that require constant psychological and behavioral adjustments. In this context, parental self-efficacy, defined as parents’ belief in their ability to positively influence their child’s development, emerges as an important psychological dimension (Albanese et al., 2019). However, according to Hamilton et al. (2015), parental self-efficacy is not an isolated factor but rather fits within the broader dimension of parenting self-regulation. Parenting self-regulation concerns parents’ perceptions of their own competence and effectiveness, understood as the ability to independently address and resolve problems, be self-determined, and modulate goals and skills over time in response to a wide variety of demands and challenges related to parenting. This construct is increasingly recognized as central not only for understanding parental flexibility but also for predicting positive outcomes for both parents and children. The concept of parenting self-regulation offers a broader perspective on parental effectiveness, highlighting four fundamental dimensions that contribute to the development of a general sense of competence and confidence in parenting. Self-efficacy refers to parents’ beliefs about their ability to effectively address and resolve specific parenting difficulties; personal agency concerns the parental locus of control, that is, the tendency to attribute children’s behaviors and outcomes to their own educational efforts rather than to random or exclusively maturational factors; self-sufficiency refers to the ability to solve problems independently and rely on one’s own resources, as well as to identify and appropriately use external resources when necessary; finally, self-management includes the processes of goal setting, self-monitoring, and self-assessment against performance criteria, which are functional to the development of parental autonomy (Hamilton et al., 2015; Matthews et al., 2022). According to the JD-R theory, effective parenting skills can be considered personal resources because they reflect positive self-evaluations of one’s ability to control and influence one’s environment successfully (Bakker et al., 2023). Albanese et al. (2019) highlighted that parental self-efficacy is often associated with better mental health in mothers and fathers, reducing the risk of anxiety, stress, and depression while promoting greater satisfaction with the parental role. 

### 1.4. The Interplay Between Job Resources, Stress, and Quiet Quitting 

In line with the JD-R theory (Bakker et al., 2023; Demerouti & Bakker, 2023), employee well-being and performance are determined by the balance of work and nonwork demands and resources. In this context, corporate welfare measures are a fundamental organizational resource, especially when personalized and tailored to employees’ specific needs (e.g., parenting support). At the same time, a parent’s ability to manage educational challenges and perceive themselves as competent acts as a crucial personal resource. These resources can protect the individual against health deterioration and negative outcomes, such as stress and quiet quitting.

Stress is not framed as an objective event, but rather as a subjective phenomenon derived from one’s perception and cognitive evaluation of events. According to Lazarus and Folkman (Lazarus, 1966; Lazarus & Folkman, 1994), a condition is considered stressful when an individual perceives it as a threat and simultaneously evaluates their coping resources as inadequate to meet the demands of their environment. The essence of the phenomenon lies in the appraisal process, whereby the psychophysical impact is determined by cognitive evaluation rather than the intrinsic properties of the external stimulus. Perceived unpredictability, a sense of uncontrollability regarding life events, and overload all contribute to the evaluation of an event (Biggs et al., 2017; Cohen et al., 1983). Although corporate welfare is a central topic in the debate on modern industrial relations, the empirical literature exploring its role is limited. Conversely, the extent to which workers perceive that their organization values their contributions and cares about their well-being has been more widely investigated. Several studies confirm the crucial role of organizational support in mitigating stress and highlight a negative relationship between the two (AlHashmi et al., 2019; Smith et al., 2022; Tetteh et al., 2020). Therefore, the perception of a supportive organization, the primary function of an effective corporate welfare system, serves as a protective factor that prevents worker distress. Conversely, parenting skills have been extensively studied in the literature and are critical to adaptation and stress prevention. They act as fundamental resources for family resilience. Parental self-efficacy is the primary cognitive and motivational factor influencing parental well-being. Empirical evidence clearly indicates a negative association between perceived self-efficacy and stress levels (Florean et al., 2025; Garcia et al., 2021; Lavenda et al., 2026; Mailey & McAuley, 2014), starting from the first weeks after childbirth (Law et al., 2019). This suggests that parents with a solid perception of competence are less vulnerable to health deterioration processes triggered by excessive environmental demands. From a physiological perspective, Buchanan et al. (2022) confirm the importance of these skills, highlighting that high levels of self-efficacy are associated with more moderate and adaptive stress response profiles, which they define as “caring but confident”. Parents who perceive themselves as competent show lower increases in cortisol and more moderate skin conductance responses when observing their children in critical situations, suggesting reduced stress and anxiety. Parental self-efficacy is especially important for parents of premature babies, children with chronic illnesses, and children with disabilities. Several studies show that these skills are an important protective factor against stress. They can be promoted by the perception of support, such as spousal or social support, or reduced by the intense burden of care these families experience (Almendingen & Pilkington, 2024; Kestler-Peleg et al., 2020; Li et al., 2022; Mundakir et al., 2025; Wu et al., 2024).

In line with the JD-R theory, perceived stress can be considered a predictor of negative organizational consequences, such as quiet quitting. Quiet quitting is a form of psychological and behavioral disengagement in which an employee deliberately limits their work effort to the minimum level necessary to avoid dismissal, without formally resigning. Although there has been rapid growth in academic interest in quiet quitting, especially in the post-pandemic period, the literature remains fragmented and lacks a universally accepted definition. Despite numerous investigations, there is still a lack of studies that systematically explore the behavioral manifestations and underlying psychological processes (Islam et al., 2026). In this context, the JD-R theory has emerged as a primary framework for understanding quiet quitting, allowing it to be viewed as both a form of disengagement and an outcome of deteriorating individual and organizational well-being (Henderson et al., 2024; Öztürk et al., 2023). Within a multidimensional framework, quiet quitting can be divided into two main dimensions: a behavioral dimension characterized by intentionally restricting commitment to only the tasks prescribed by the contract and refusing extra-role activities (e.g., organizational citizenship behaviors); and an emotional/affective dimension reflecting psychological detachment combined with satisfaction or personal comfort in maintaining this minimum level of contribution (Patel et al., 2025). Quiet quitting can also be interpreted as an adaptive response to work-family conflict. In this case, workers establish clear boundaries between their professional and private lives to protect the time and energy they devote to caring for their families. Thus, the practice of “doing the minimum” is not necessarily hostile toward the company but rather a strategy for managing work and nonwork demands to achieve a better work–life balance (Bernuzzi et al., 2025). The authors suggest that quiet quitting is driven by a lack of organizational and personal resources needed to cope with work demands. Possible antecedents include the perception of support and the presence of policies that promote work–life balance. These factors act as protective measures, mitigating stress and preserving employee engagement. In fact, several studies highlight that a strong organizational support system and organizational policies are a powerful inhibitor of quiet quitting (Geng et al., 2026; Haar et al., 2026; Nimmi et al., 2024). Conversely, the absence of targeted welfare policies, such as support systems for new parents or provisions for health and mental well-being, is perceived as a breach of the psychological contract, which fosters the propensity for quiet quitting (Dilchert et al., 2025). Therefore, organizations that fail to mitigate work-family conflict or show a lack of concern for employees’ overall well-being may cause individuals to become disengaged, seeking personal space to pursue self-development and family care. One interesting finding is the impact of caregiving responsibilities. Unexpectedly, childcare responsibilities and marital status are protective factors against quiet quitting. Employees with caregiving responsibilities report lower levels of quiet quitting. This suggests that the stability provided by the family unit and the sense of purpose associated with parenthood promote greater conscientiousness and dedication in a professional context (Dilchert et al., 2025). However, the effectiveness of these protective factors often depends on parents’ perceived ability to exert active control over their environment. In this sense, self-efficacy can enable workers to cope with work challenges without experiencing stress. Furthermore, empirical evidence confirms a positive association between perceived stress levels and the tendency to quietly quit (Ardıç & Erişen, 2025; Dilekçi et al., 2025). This dynamic encompasses several dimensions of stress, including conscious stress (i.e., experienced when a discrepancy arises between one’s professional values and the conditions in which one is required to work) (Danacı et al., 2026) and technostress (i.e., triggered by technology-related demands that exceed one’s ability to cope) (El-Sayed et al., 2026). Furthermore, stress frequently serves as a mediator between work (e.g., organizational caring climate) and nonwork (e.g., personal caregiver identity) demands/resources and the intention to quit (Boz, 2026; Dong, 2026).

Therefore, preventing quiet quitting seems to require transitioning to a more “human-centered” approach to human resource management. This approach would systematically integrate support for parenthood and psychophysical well-being as pillars of organizational strategy.

### 1.5. Aims of the Study 

The present study has a twofold aim. First, it seeks to evaluate the psychometric properties of the Perceived Corporate Welfare Scale through confirmatory factor analysis (CFA) to confirm the scale’s single-factor structure and validity. All the participants in this study were considered for the first objective. Second, in line with JD-R theory, the study aims to explore the associations among perceived corporate welfare (as an organizational resource), parenting self-regulation (as a personal resource), perceived stress, and quiet quitting, hypothesizing that perceived stress mediates the relationship between these resources and quiet quitting. Additionally, the study aims to explore the role of parenthood as a moderator in the relationship between perceived stress and quiet quitting, with the aim of identifying any differences in perceptions between mothers and fathers. Only parents were considered for this second objective. The present study is intended to provide HR managers and top management with twofold benefits. First, it provides a valid and reliable measurement tool for evaluating the effectiveness of corporate welfare policies. Second, the empirical evidence may offer strategic insights for designing parenting support initiatives tailored to specific needs rather than a one-size-fits-all approach.

## 2. Materials and Methods

### 2.1. Participants and Procedure

The study was grounded in a cross-sectional research design, under which data were collected concurrently and involved participants from different organizations in Italy. Participants were eligible if they were 18 or older and employed. The data were collected in 2025, after the COVID-19 pandemic ended. A total of 788 workers were recruited via snowball sampling and invited to participate in the study. This study is part of a larger project focused on promoting well-being and performance in organizational contexts by exploring the role of work and nonwork-related demands and resources, including those related to parenting.

Participants were administered an online self-report questionnaire using Google Forms. Once they provided written informed consent (including assurances of anonymity and the treatment of the data), they completed the questionnaire. The study was performed in line with the principles of the Declaration of Helsinki. The project was approved by the Psychological Research Ethics Committee (Area 17), Department/Section of Psychology—University of Padua, Italy (protocol n. 616-a, approved on 17 May 2024; update protocol n. 754-a, approved on 24 July 2024).

Table 1 presents the participants’ socio-demographic characteristics. About half of the participants were between 18 and 39 years old (52.5%), approximately one in five participants was between 40 and 49 years old (20.9%), and between 50 and 59 years old (20.6%), and the remaining 6.0% were over 60 years old. Regarding education, more than one-third of workers held a university degree (38.7%) and a high school diploma (38.7%), 10.8% held an elementary/middle school diploma, 10.3% held a post-graduate degree (e.g., PhD), and the remaining 1.5% indicated “Other”. Most participants were employed in paid work (85.5%); 11.2% were freelance; 3.3% indicated “Other”. The majority had an open-ended contract (65.4%); 17.9% had a fixed-term contract; 11.5% did not respond; and 5.2% indicated “Other”. Three-quarters of the participants reported working full-time (40 h/week; 75.0%), whereas 20.8% worked part-time (between 20 and 36 h/week); 4.2% indicated “Other”. Regarding marital status, about half of the participants reported being married or cohabiting (54.4%), 35.8% reported being single or unmarried, and 8.0% reported being divorced or separated; the remaining 1.8% indicated “Other”. About half of the participants were parents of at least one child (mothers = 29.6%; fathers = 26.9%), and the remaining 43.5% reported they are not parents. Regarding the number of children, 46.3% of parents report having one child, 44.7%% report having two children, 8.3% report having three or more children, and 0.7% did not respond.

### 2.2. Measures

Perceptions of corporate welfare were assessed with the scale proposed by Rapisarda et al. (2025). The authors proposed a new scale designed to assess employees’ perceptions of critical elements that contribute to the effectiveness of a welfare plan. Specifically, the objective was to provide an initial contribution to the evaluation of the factorial structure of the new scale. The authors emphasize the added value of a scale designed to measure employee perceptions of aspects such as the communication of the welfare plan, adaptability to employees’ needs, and the sustainability of such measures, rather than focusing solely on satisfaction and awareness of benefits. After reviewing the literature and consulting with experts, the authors proposed nine items on a 5-point Likert scale. The initial results retained all proposed items, confirmed the scale’s single-factor structure, and demonstrated good reliability. Our final version (named Perceived Corporate Welfare Scale) confirms a one-factor structure and comprises nine items, such as “Welfare measures are identified by my Organization on the basis of an analysis of my specific needs (e.g., support with caregiving responsibilities)”. The response scale ranged from 1 (strongly disagree) to 5 (strongly agree). The Cronbach’s alpha for the scale was 0.93 for all participants and 0.94 for parents.

Parenting self-regulation was assessed using the “Me as a Parent” Questionnaire (Hamilton et al., 2015). The scale is composed of 16 items equally distributed in four dimensions: self-efficacy (e.g., “I have confidence in myself as a parent”), personal agency (e.g., “I often feel helpless about my child’s behavior”), self-sufficiency (e.g., “I know how to solve most problems that arise with parenting”), and self-management (e.g., “I meet my expectations for providing emotional support for my child”). Only the parents were asked to respond on a scale ranging from 1 (strongly disagree) to 5 (strongly agree). Cronbach’s alpha for the overall scale was 0.87.

Perceived stress was assessed using the negative subscale of the Italian Perceived Stress Scale (I-PSS-10) (Mondo et al., 2021). The subscale is composed of six items, such as “In the last month, how often have you felt unable to control the important things in your life?”. The response scale ranged from 0 (never) to 4 (very often). The Cronbach’s alpha for the scale was 0.86 for both all participants and parents.

Quiet quitting was measured using the Multidimensional Quiet Quitting Scale (MQQS) (Patel et al., 2025). The scale consists of 11 items and comprises two dimensions: behavioral aspects of quiet quitting (5 items, e.g., “I do only the work I’m specifically asked to do; just enough to not lose my job”) and emotional aspects of quiet quitting (6 items, e.g., “Worries weigh heavily on my mind, when going above and beyond in my work”). The response scale ranged from 1 (strongly disagree) to 5 (strongly agree). Cronbach’s alpha for the overall scale was 0.86 for all participants and 0.89 for parents. 

Finally, socio-demographic information (parenthood, age, education, occupation, work contract, work time, marital status, number of children) was assessed.

### 2.3. Data Analysis

Harman’s single-factor test was used to assess common method bias (CMB). If only one factor emerges from the factor analysis or if one factor accounts for most of the variance among the variables (i.e., more than 50% of the variance), there is a risk of CMB (Podsakoff et al., 2003, 2024).

A series of confirmatory factor analyses (CFA) using the robust maximum likelihood method (MLM) was carried out to evaluate the psychometric properties of the Perceived Corporate Welfare Scale and, more generally, the self-report instruments administered in the study for both all participants (*N* = 788) and parents (*n* = 445). In these models, each dimension of perceived corporate welfare and perceived stress was measured by the respective items. To estimate fewer parameters and improve the reliability of observed indicators, parcels were created using the internal-consistency approach, using facets as the grouping criteria. This approach has two main benefits. First, it keeps the construct’s multidimensional nature clear. Second, it allows the unique component of a facet to relate to other constructs in the model (Little et al., 2002). Specifically, four parcels of scale items measured parenting self-regulation (i.e., self-efficacy, personal agency, self-sufficiency, and self-management), and two parcels measured quiet quitting (i.e., behavioral and emotional quiet quitting). We used the chi-square (χ^2^) test to assess the model’s adequacy. A non-significant χ^2^ indicated an acceptable model fit. However, because the χ^2^ is affected by sample size, we considered additional fit indices: the comparative fit index (CFI), the Tucker–Lewis Index (TLI), the root-mean-square error of approximation (RMSEA), and the standardized root-mean-square residual (SRMR). Values ≥ 0.90 for CFI and TLI and ≤ 0.08 for RMSEA and SRMR indicated an acceptable model fit (Brown, 2015). We also assessed the composite reliability (CR) and the average variance extracted (AVE) indices, whose values ≥ 0.70 and ≥0.50, respectively, are considered satisfactory (Bagozzi & Yi, 2012).

Descriptive statistics and Pearson’s correlation were assessed to preliminarily assess the relationships among the study variables. Next, a structural equation model with observed variables (i.e., path analysis) was estimated to investigate the relationship among perceived corporate welfare, parenting self-regulation, and quiet quitting and to test the role of perceived stress as a mediator of these relationships. Furthermore, we tested the role of parenthood as a moderator of the relationship between perceived stress and quiet quitting. Bootstrap methods at 95% confidence intervals were used. A statistically significant effect is supported if a confidence interval does not contain zero (MacKinnon et al., 2012). All the analyses were performed using SPSS 29 (IBM Corp, 2023) and jamovi software version 2.7 (The jamovi project, 2024), built on top of the R statistical language version 4.5 (R Core Team, 2023).

## 3. Results

### 3.1. Common Method Bias Assessment and Psychometric Properties of the Self-Report Instrument

Harman’s single-factor test was used to explore the CMB of the data. For all participants (*N* = 788), perceived corporate welfare, perceived stress, and quiet quitting items were included in the unrotated exploratory factor analysis. The number of factors with an eigenvalue greater than 1 was three, and the explained variance of the first factor was 26.10%, lower than the critical criterion of 50%. In addition, the results of a CFA combining all the items also showed a poor fit to the data (χ^2^(299) = 5863, *p* < 0.001; CFI = 0.477; TLI = 0.341; RMSEA = 0.154; SRMR = 0.189). Therefore, there was no apparent CMB in the data of this study. Similar results were found for parents. In addition to the items that were previously analyzed, those related to parenting self-regulation were also included. The number of factors with an eigenvalue greater than 1 was four, and the explained variance of the first factor was 20.92%. The results of a CFA combining all the items also showed a poor fit to the data (χ^2^(819) = 7055, *p* < 0.001; CFI = 0.355; TLI = 0.322; RMSEA = 0.131; SRMR = 0.176). Therefore, also for parents, there was no apparent CMB in the data of this study.

The hypothesized three-factor CFA model for the self-report instrument showed a good fit to data for all participants (χ^2^(116) = 413.40, *p* < 0.001; CFI = 0.952; TLI = 0.944; RMSEA = 0.057; SRMR = 0.035). Similar results emerged for the hypothesized four-factor CFA model among parents (χ^2^(183) = 419.70, *p* < 0.001; CFI = 0.950; TLI = 0.942; RMSEA = 0.054; SRMR = 0.047). Furthermore, the CR and AVE values for all dimensions reached satisfactory values for both all participants and parents. The CR ranges from a minimum of 0.805 to a maximum of 0.940, and the AVE ranges from a minimum of 0.501 to a maximum of 0.724. Overall, the self-report questionnaires used in this study showed good psychometric properties.

### 3.2. Psychometric Properties of the Perceived Corporate Welfare Scale

The one-factor model showed a good fit to the data (*N* = 788) − χ^2^(27) = 185.8, *p* < 0.001; CFI = 0.955; TLI = 0.941; RMSEA = 0.086; SRMR = 0.037. Table 2 shows standardized path coefficients, which were all significant and greater than 0.60. Furthermore, CR and AVE reached satisfying values for the scale (CR = 0.931; AVE = 0.600). Therefore, we considered the measurement model validity appropriate. The list of items on the scale can be found in Appendix A.

Table 3 presents the means, standard deviations, and correlations among perceived corporate welfare, perceived stress, and quiet quitting. 

### 3.3. The Moderated Mediation Model

While the initial analyses were conducted on the full group of participants (*N* = 788), the moderated mediation model was tested using only the subsample of working parents (*n* = 445). This targeted approach allows for a deeper examination of the psychological mechanisms specific to the work-family interface, where the impact of supportive policies is most salient.

Table 4 presents the means, standard deviations, and correlations among the measured psychological dimensions. Perceived corporate welfare was positively correlated with parenting self-regulation (*r* = 0.209, *p* < 0.001) and negatively correlated with perceived stress (*r* = −0.240, *p* < 0.001) and quiet quitting (*r* = −0.164, *p* < 0.001). Parenting self-regulation was also negatively correlated with perceived stress (*r* = −0.290, *p* < 0.001) and quiet quitting (*r* = −0.246, *p* < 0.001). Finally, perceived stress was positively correlated with quiet quitting (*r* = 0.359, *p* < 0.001).

**Figure 1:**
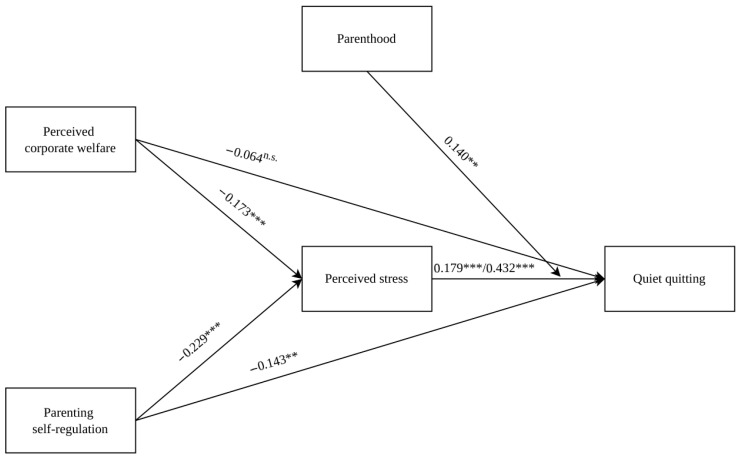
The path analysis model. Note. Standardized coefficients are reported. The effects of perceived stress on quiet quitting are first presented for mothers, then for fathers. ^n.s.^ = not significant; ** *p* < 0.01; *** *p* < 0.001.

A structural equation model with observed variables was implemented to investigate the relationship among perceived corporate welfare, parenting self-regulation, and quiet quitting, and to test the mediating role of perceived stress as well as the moderating role of parenthood (Figure 1).

As shown in Table 5, perceived corporate welfare and parenting self-regulation were negatively associated with perceived stress (β = −0.173, *p* < 0.001; β = −0.229, *p* < 0.001). The association between perceived corporate welfare and quiet quitting was not significant (β = −0.064, *p* > 0.05), whereas the association between parenting self-regulation and quiet quitting was significant (β = −0.143, *p* < 0.01). Perceived stress among mothers and fathers was positively associated with quiet quitting, controlling for the effects of perceived corporate welfare and parenting self-regulation (β = 0.179, *p* < 0.001; β = 0.432, *p* < 0.001). Furthermore, regarding the moderating effect of parenthood, the results showed that the interaction term was positive and significant (β = 0.140, *p* < 0.01), indicating a difference between mothers and fathers in the relationship between perceived stress and quiet quitting. Specifically, the association between perceived stress and quiet quitting appeared stronger among fathers (β = 0.432, *p* < 0.001) than among mothers (β = 0.179, *p* < 0.001).

Finally, results suggest that parenthood moderates the indirect effects of perceived corporate welfare and parenting self-regulation on quiet quitting through perceived stress (Table 6). Specifically, both indirect effects were stronger among fathers (β = −0.075, 95% CI = −0.1200, −0.0297; β = −0.099, 95% CI = −0.2630, −0.0874) than among mothers (β = −0.031, 95% CI = −0.0576, −0.0049; β = −0.041, 95% CI = −0.1273, −0.0145).

## 4. Discussion and Conclusions

The first aim of the present study was to test the psychometric properties of the Perceived Corporate Welfare Scale among all participants. The second aim was to explore the roles of perceived corporate welfare and parenting self-regulation as organizational and personal resources, respectively, in protecting against perceived stress and, consequently, quiet quitting. Furthermore, the differences between mothers and fathers were examined. The findings support the unidimensional structure of the scale, retaining all nine items, and its reliability and validity. Regarding the hypothesized model, in line with JD-R theory, perceived corporate welfare and parenting self-regulation function as resources and are negatively associated with perceived stress. Perceived stress fully mediates the relationship between perceived corporate welfare and quiet quitting and partially mediates the relationship between parenting self-regulation and quiet quitting. The results further show that this relationship is moderated by parental status: fathers’ perceived stress has a stronger association with quiet quitting than mothers’ does. Consequently, the indirect effects are also stronger for fathers.

The moderation analysis is particularly interesting and merits some initial considerations. First, the lower level of quiet quitting among mothers may stem from the pressure exerted by the stereotypical “ideal worker” view (Williams, 2000). Good workers are fully immersed in their work and either have no children or have someone to take care of them. Working mothers who perceive this prejudice (Hampson, 2018) may compensate for it by engaging in overperformance behaviors. This is not necessarily greater engagement, but rather a defensive strategy to refute the “bad worker” stereotype of mothers by adhering to high work standards. A second possible explanation lies in labor market asymmetries. The literature indicates that women face greater obstacles to career advancement, external mobility, and access to equally remunerated positions (Blau & Kahn, 2017). For mothers, quiet quitting may pose a greater economic and professional risk than it does for fathers. A lower perception of equivalent or superior employment alternatives may force mothers to maintain high performance standards to preserve their acquired stability, rendering psychological disinvestment a “luxury” many feel they cannot afford. 

This study contributes to the JD-R theory literature by offering theoretical insights and practical applications. First, the direct relationship between work and nonwork resources and stress was examined, revealing a negative association. JD-R theory originally predicts that job resources are negatively related to strain through their relationship with work engagement and that they buffer the positive impact of job demands on strain (Bakker & Demerouti, 2024). However, several meta-analytic studies have reported a direct negative relationship between job resources and burnout (e.g., Lesener et al., 2019). The results of the present study seem to confirm the direct relationship between resources and worker health. Additionally, while the JD-R theory has traditionally emphasized job demands and resources as primary determinants of well-being, recent developments underscore the importance of incorporating demands and resources from other life domains (Bakker et al., 2023). This study advances the theory by incorporating personal resources related to the family dimension. Specifically, considering parenting self-regulation (i.e., the perception by parents of their autonomy and capability to face the challenges of parenthood, assess their own resources and need for external resources, and maintain a high internal locus of control) as a personal resource reveals that the self-regulation skills developed within the family interact with organizational resources, such as perceived corporate welfare, and are associated with reduced perceived stress and quiet quitting. Thus, the current study suggests that understanding organizational well-being requires a holistic consideration of the individual. Finally, to the best of our knowledge, this is one of the first studies to consider corporate welfare (i.e., the perception that an organization plans sustainable, tailored welfare measures, such as family-friendly policies, and monitors, communicates, and makes them easily accessible over time) as an organizational resource (Dal Corso, 2024; Dal Corso & Rapisarda, in press) associated with the promotion of health (Demerouti & Bakker, 2023). 

The present study offers practical contributions to organizations. First, the proposed Perceived Corporate Welfare Scale is an innovative strategic tool for assessing perceptions of corporate welfare. Its concise nature also meets the need for agile, frequent monitoring by overcoming the limitations of overly burdensome surveys, which often reduce response rates. Several studies (Jayanthi & Ilangovan, 2019; Sasirekha et al., 2021; Vinitha et al., 2020) have reported on tools that measure employee satisfaction with specific corporate welfare programs, including both tangible and intangible programs. However, a tool that can measure the effectiveness of a corporate welfare plan by assessing its ability to meet employees’ specific needs during the design phase and monitor its implementation and dissemination would be useful (CENSIS, 2026). Indeed, there is a clear need to move beyond standardized welfare approaches toward highly personalized plans that address specific parenting needs (Rapisarda et al., 2025). This personalization should extend beyond external resources and be integrated into a multi-level prevention strategy. Primary interventions should redesign work to reduce demands, for example, by offering flexible hours. Secondary interventions should strengthen personal resources, such as parental self-regulation, through training programs (e.g., parent education, skill development, social support activities, anxiety and stress management) (Rapisarda et al., 2024b; Waid et al., 2025). Digital interventions offer innovative solutions by increasing parents’ access to information, improving their compliance with the intervention, and facilitating access to context-specific resources (Lin-Lewry et al., 2024; Rapisarda et al., 2024a). Finally, tertiary interventions, such as professional psychological support, can help address stress-related symptoms and counteract quiet quitting behaviors. Therefore, it is important for organizations to promote family-friendly policies and support systems. These measures are effective when they combine formal policies related to flexibility, leave, care support, and career development with informal supports, such as emotional, instrumental, and social support provided by supervisors and colleagues. These interventions can improve career outcomes, such as intention to stay and career success; job outcomes, such as hedonic and eudemonic well-being; and work-family outcomes, such as work-life balance and work-family conflict (Blom et al., 2025). However, merely implementing these strategies does not guarantee success. Often, employees—especially parents—face the risk of work-family backlash (Perrigino et al., 2018) for using family-friendly policies and supports. This risk can lead mothers and fathers to use such policies less frequently than people without children do. Therefore, for family-friendly policies to be effective, organizations must promote a family-friendly culture where support for families is legitimized by leadership’s daily behavior and corporate values are shared throughout the workplace.

Nevertheless, this study has several limitations. First, the research design is cross-sectional. Since the data on the predictive variables and outcomes were collected simultaneously, it is not possible to establish temporal precedence. While the proposed theoretical model is theoretically supported by the literature, future studies should use longitudinal designs to confirm the stability of the observed relationships over time and reduce the risk of ambiguous interpretations. In addition, parcels have been created. Although they offer advantages in parsimony and model stability, some argue that using parcels can distort reality or impose an arbitrary false structure. However, a pragmatic perspective supports the thoughtful use of parcels, viewing them as a means to achieve advantages in terms of psychometric and fit characteristics (Little, 2013; Little et al., 2002). Second, exclusively using self-report instruments may be subject to social desirability bias or distortions due to momentary affective states. Future research would benefit from a multi-source approach, such as integrating assessments from supervisors or colleagues, or using objective indicators to increase the validity of the measures. Third, the group of participants in this study is homogeneous across socio-demographic variables, limiting the generalizability of the results. Specifically, the prevalence of high educational attainment, the uniformity in contract type (i.e., open-ended contract), and working hours (i.e., full-time) limit generalizability to workers with different backgrounds. Additionally, most participants reported being married. Since nonwork demands and resources vary across single, cohabiting, and separated individuals, the homogeneity of the group of participants limits the generalizability of the results to other family configurations. Therefore, future studies should include a more heterogeneous sample, including diverse work situations and family contexts, to test the relationships identified in this study across different segments of the working population. Specifically, it would be interesting to investigate the subpopulation of younger working parents. In addition, it would be useful not only to detect the presence of children but also to specify their age and distinguish among different care demands (e.g., early childhood versus school-age). This is because the intensity and nature of work-family conflict vary significantly depending on the stage of the children’s life cycle. Additionally, including other work and nonwork resources and demands (e.g., supervisor work-life role modeling, perceived support from a partner, social support, mental labor, care burden, perceived stigma, labor market opportunities) would help better understand which dimensions contribute to parents’ individual and organizational well-being, as well as the differences between the roles of fathers and mothers. Due to the current theoretical fragmentation in the debate on quiet quitting (Islam et al., 2026), future research should continue to explore which resources and demands serve as protective or risk factors in the development of this phenomenon. Integrating JD-R theory with the Conservation of Resources theory (Demerouti, 2025; Hobfoll et al., 2018), a framework used to explain quiet quitting, appears promising. This integration could provide a more comprehensive interpretive lens for understanding the phenomenon and its behavioral manifestations. Finally, to overcome a strictly individualistic view, multilevel research designs should be adopted. These designs would enable researchers to examine crossover effects and understand how one partner’s work characteristics or well-being influences the other’s well-being and performance. Considering couples, rather than individuals, would allow us to capture the mutual influence between individual and relational dimensions, offering a more systemic and realistic view of organizational and personal dynamics.

Work and nonwork resources may act synergistically as powerful protective factors capable of mitigating stress. This, in turn, may prevent negative outcomes such as quiet quitting. Therefore, promoting an organizational culture that recognizes and values the parenting role is a strategic decision for maintaining employee engagement and well-being.

## Figures and Tables

**Table 1:** Participants’ socio-demographic characteristics.

Socio-Demographic	Frequence	Percentage
Age		
Between 18 and 39 years	414	52.5%
Between 40 and 49 years	165	20.9%
Between 50 and 59 years	162	20.6%
60 and over	47	6.0%
Education		
Elementary/Middle school diploma	85	10.8%
High school diploma	305	38.7%
University degree	305	38.7%
Post-graduate degree	81	10.3%
Other	12	1.5%
Occupation		
Employed in paid work	674	85.5%
Freelance	88	11.2%
Other	26	3.3%
Work contract		
Open-ended contract	515	65.4%
Fixed-term contract	141	17.9%
Other	41	5.2%
Missing data	91	11.5%
Work time		
Full-time	591	75.0%
Part-time	164	20.8%
Other	33	4.2%
Marital status		
Married or cohabiting	429	54.4%
Divorced or separated	63	8.0%
Single or unmarried	282	35.8%
Other	14	1.8%
Parenthood		
Non-parent	343	43.5%
Mother	233	29.6%
Father	212	26.9%
Number of children *		
One	206	46.3%
Two	199	44.7%
Three or more	37	8.3%
Missing data	3	0.7%

Note. * Only parents’ responses were considered.

**Table 2:** Perceived Corporate Welfare Scale’s standardized coefficients.

Items	Standardized Coefficients
Item 1	0.712
Item 2	0.748
Item 3	0.800
Item 4	0.608
Item 5	0.834
Item 6	0.860
Item 7	0.898
Item 8	0.814
Item 9	0.870

**Table 3:** Means, standard deviations, and correlations among perceived corporate welfare, perceived stress, and quiet quitting.

Variables	*M*	*SD*	1	2	3
1. Perceived corporate welfare	3.001	0.906	-		
2. Perceived stress	1.568	0.877	−0.252 ***	-	
3. Quiet quitting	2.338	0.828	−0.144 ***	0.326 ***	-

Note. *M* = mean; *SD* = standard deviations; *** *p* < 0.001.

**Table 4:** Means, standard deviations, and correlations among the study variables.

Variables	*M*	*SD*	1	2	3	4
1. Perceived corporate welfare	3.055	0.942	-			
2. Parenting self-regulation	3.887	0.522	0.209 ***	-		
3. Perceived stress	1.466	0.855	−0.240 ***	−0.290 ***	-	
4. Quiet quitting	2.258	0.851	−0.164 ***	−0.246 ***	0.359 ***	-

Note. *M* = mean; *SD* = standard deviations; *** *p* < 0.001.

**Table 5:** Coefficients of the moderated mediation model.

Antecedents	Outcomes									
	Perceived Stress					Quiet Quitting				
	*B*	*SE*	β	*p*	95% CI	*B*	*SE*	β	*p*	95% CI
Perceived corporate welfare	−0.157	0.041	−0.173	0.001	[−0.2425; −0.0739]	−0.058	0.041	−0.064	0.152	[−0.1422; 0.0294]
Parenting self-regulation	−0.375	0.074	−0.229	0.001	[−0.5298; −0.2153]	−0.232	0.075	−0.143	0.002	[−0.3923; −0.0865]
Parenthood	−0.264	0.077	−0.155	0.001	[−0.4152; −0.1132]	−0.327	0.150	−0.048	0.029	[−0.6213; −0.0329]
Perceived stress—Mother						0.174	0.046	0.179	0.001	[0.0413; 0.3022]
Perceived stress—Father						0.453	0.046	0.432	0.001	[0.2899; 0.6235]
Perceived stress X Parenthood						0.279	0.088	0.140	0.002	[0.1054; 0.4529]

**Table 6:** Conditional indirect effects of perceived corporate welfare and parenting self-regulation on quiet quitting through perceived stress among mothers and fathers.

Moderator Levels	Indirect Effect	Standardized Indirect Effect	Bootstrap 5000 Times 95% CI	
			CI Lower	CI Upper
Mother	WELF → STRESS → QQ	−0.031	−0.0576	−0.0049
Father	WELF → STRESS → QQ	−0.075	−0.1200	−0.0297
Mother	PSR → STRESS → QQ	−0.041	−0.1273	−0.0145
Father	PSR → STRESS → QQ	−0.099	−0.2630	−0.0870

Note. WELF = perceived corporate welfare; PSR = parenting self-regulation; STRESS = perceived stress; QQ = quiet quitting; CI = confidence interval.

## Data Availability

The original contributions presented in the study are included in the article, and further inquiries can be directed to the corresponding author.

## Notes

1 Presidential Decree No. 917 of 22 December 1986. Approvazione del testo unico delle imposte sui redditi [Approval of the Italian Tax Consolidated Text]. Gazzetta Ufficiale No. 302 (31 December 1986). Art. 51, 100.

2 Law No. 208 of 28 December 2015. Disposizioni per la formazione del bilancio annuale e pluriennale dello Stato (Legge di Stabilità 2016) [Provisions for the formation of the annual and multi-year State budget (Stability Law 2016)]. Gazzetta Ufficiale No. 302 (30 December 2015).

3 Law No. 232 of 11 December 2016. Bilancio di previsione dello Stato per l’anno finanziario 2017 e bilancio pluriennale per il triennio 2017-2019 [State budget for the 2017 financial year and multi-year budget for the 2017–2019 triennium]. Gazzetta Ufficiale No. 297 (21 December 2016).

## Abbreviations

The following abbreviations are used in this manuscript:

AVE	Average Variance Extracted
CFA	Confirmatory Factor Analysis
CFI	Comparative Fit Index
CI	Confidence Interval
CMB	Common Method Bias
CR	Composite Reliability
JD-R	Job Demands-Resources
M	Mean
PSF	Parenting self-regulation
QQ	Quiet quitting
RMSEA	Root-Mean-Square Error of Approximation
SD	Standard Deviation
SRMR	Standardized Root-Mean-Square Residual
STRESS	Perceived stress
TLI	Tucker–Lewis Index
WELF	Perceived corporate welfare

## Appendix A. Perceived Corporate Welfare Scale

Please read the following statements carefully regarding corporate welfare measures (i.e., the set of initiatives, goods, and services implemented by the Organization to promote the well-being of individuals). Please indicate your level of agreement or disagreement using a response scale ranging from 1 = Strongly disagree to 5 = Strongly agree.

*Le chiediamo di leggere attentamente le seguenti affermazioni relative alle misure di welfare aziendale, intese come l’insieme di iniziative, beni e servizi attivati dall’Organizzazione per promuovere il benessere delle persone. Le chiediamo di indicare il Suo grado di accordo o di disaccordo attraverso una scala di risposta che va da 1 = Fortemente in disaccordo a 5 = Fortemente d’accordo.*

Strongly disagree *Fortemente in* *disaccordo*	Disagree *In disaccordo*	Mixed feelings *Né in disaccordo* *né d’accordo*	Agree *D’accordo*	Strongly agree *Fortemente* *d’accordo*
1	2	3	4	5

**Items**
1. Welfare measures are identified by my Organization on the basis of an analysis of my specific needs (e.g., support with caregiving responsibilities) *1. Le misure di welfare sono individuate dalla mia Organizzazione sulla base dell’analisi delle mie specifiche necessità (es. supporto nei carichi di cura)*
2. Welfare measures proposed by my Organization address my needs (e.g., flexible working hours) *2. Le misure di welfare proposte dalla mia Organizzazione rispondono ai miei bisogni (es. orari flessibili)*
3. My Organization systematically monitors whether the welfare measures provided are adequate to meet my needs *3. L’Organizzazione in cui lavoro monitora in modo costante se le misure di welfare proposte siano adeguate ai miei bisogni*
4. I am aware of the welfare measures offered by my Organization *4. Sono a conoscenza delle misure di welfare proposte dalla mia Organizzazione*
5. My Organization effectively implements what it communicates regarding welfare policies *5. La mia Organizzazione mette in pratica ciò che comunica in tema di welfare*
6. Within my Organization, I can easily access welfare measures that support my well-being (e.g., by promoting a better work-life balance) *6. Nella mia Organizzazione posso usufruire facilmente delle misure di welfare a sostegno del mio benessere (es. favorendo un miglior equilibrio tra vita e lavoro)*
7. Welfare measures proposed by my Organization demonstrate its concrete commitment to sustainability *7. Le misure di welfare proposte dalla mia Organizzazione sono la dimostrazione del suo impegno concreto in termini di sostenibilità*
8. Welfare measures identified by my Organization contribute to reducing pay gaps and promoting gender equity within my work environment (e.g., supporting parenthood) *8. Le misure di welfare individuate dalla mia Organizzazione contribuiscono al contrasto del divario retributivo e alla promozione dell’equità di genere nel mio contesto lavorativo (es. sostenendo la genitorialità)*
9. Welfare measures adopted in my Organization foster a corporate culture oriented toward employee well-being and the care of their family members *9. Le misure di welfare adottate nella mia Organizzazione promuovono una cultura aziendale orientata al benessere dei/delle dipendenti e alla cura dei loro familiari*
Note. This scale may be freely copied as long as (a) authors are credited, (b) no changes are made, and (c) it is not sold. Anyone interested in using the scale should notify the corresponding author.
